# Accidental Aluminum Phosphide Intoxication Successfully Treated with Hyperbaric Oxygen Therapy: A Case Report

**DOI:** 10.3390/toxics12040272

**Published:** 2024-04-05

**Authors:** Michal Hájek, Dittmar Chmelař, Jakub Tlapák, Veronika Rybárová, Peter Ondra, Vladimír Halouzka

**Affiliations:** 1 Centre of Hyperbaric Medicine, Ostrava City Hospital, 72880 Ostrava, Czech Republic; michalhajek@email.cz; 2 Institute of Laboratory Medicine, Institute of Microbiology, Faculty of Medicine, University of Ostrava, 70300 Ostrava, Czech Republic; 3 Centre for Hyperbaric Medicine of Faculty of Medicine University of Ostrava and Ostrava City Hospital, 70300 Ostrava, Czech Republic; 4 The Institute of Aviation Medicine, 16000 Prague, Czech Republic; 5 Faculty of Biomedical Engineering, Czech Technical University in Prague, 16000 Prague, Czech Republic; 6 Department of Forensic Medicine and Medicolegal Expertises, Jessenius Faculty of Medicine, Comenius University, University Hospital, 036 01 Martin, Slovakia; 7 Department of Forensic Medicine and Medical Law, University Hospital Olomouc and Faculty of Medicine and Dentistry, Palacky University Olomouc, 77900 Olomouc, Czech Republic

**Keywords:** case reports, phosphine gas, aluminum phosphide poisoning, lactic acidosis, hyperbaric oxygen therapy

## Abstract

The most common pesticide agents are organophosphates and phosphides, aluminum phosphide (ALP) in particular. ALP is a major cause of suicidal poisoning in many countries. In other countries, the problem of accidental, mainly occupational-related, poisoning is also real and actual. Almost two thirds of individuals in poisoning cases have died. This case report describes a case of a patient with accidental ALP intoxication. The origin of the poisoning was the fumigation of stored grain in an agricultural building adjacent to the building in which patient was temporarily housed, while both buildings were connected by an underground corridor, through which the released poison gas penetrated. The case was originally presented by the rescuers as well as healthcare professionals of the local hospital as carbon monoxide intoxication, which has a similar symptomatology as ALP intoxication. The patient was treated comprehensively, including using the HBOT method, which is very unique in the case of phosphine intoxication in human medicine, with an excellent final clinical outcome. This was the first described case of HBOT for ALP intoxication in clinical medicine, although the HBOT indication itself became a coincidence in this case. Further studies must be undertaken to demonstrate the effectiveness of HBOT in treating patients with ALP poisoning.

## 1. Introduction

Every year, about 300,000 people die because of pesticide poisoning worldwide. The most common pesticide agents are organophosphates and phosphides, aluminum phosphide (ALP) in particular [1]. ALP is one of the leading causes of suicidal poisoning in many countries [2,3]. In a group of patients hospitalized in a specialized clinic within 1 year for different intoxications, the most common (76%) poisoning substance was aluminum phosphide. Almost two thirds of those involved in poisoning cases died [4]. In other countries, the issue of unintentional poisonings, particularly those occurring in occupational settings, is of current relevance. In particular, container shipping workers are at risk; where the so-called fumigation of cargo spaces on the ship is carried out, workers and handlers at port trans-shipment yards are also at risk [5,6,7].

ALP is a cheap solid fumigant and a highly toxic pesticide which is commonly used for grain preservation. ALP can be synthesized as dark gray or dark yellow crystals and can take the form of tablets, pellets, granules, or dust. It is marketed as 3 g tablets consisting of ALP (56%) and carbamate (44%) [1]. Upon contact with moisture in the environment, ALP undergoes a chemical reaction that produces phosphine gas, which is the active pesticidal component [8]. A lethal dose of ALP is 1–1.5 g. Deaths are reported with doses of 150–500 mg. Mortality with ALP poisoning is very high, ranging from 37% to 100% [9,10].

The National Institute for Occupational Safety and Health (NIOSH) has established a limit for occupational exposure to phosphine gas at 0.3 ppm and has deemed it “immediately dangerous to life or health” at 50 ppm or more [11,12].

ALP-induced toxicity is caused by the liberation of phosphine gas, which causes cell hypoxia due to the inhibition of oxidative phosphorylation leading to circulatory failure [1]. Phosphine leads to the non-competitive inhibition of mitochondria cytochrome oxidase, blocking the electron transfer chain and oxidative phosphorylation, producing an energy crisis in the cells [10].

In its management, the main objective is to provide effective oxygenation, ventilation, and circulation until phosphine is excreted. All patients of severe ALP poisoning require continuous invasive hemodynamic monitoring and early resuscitation with fluid and vasoactive agents. Many therapeutic agents have been tried in experimental animal studies, such as N-acetylcysteine and GSH, hydroxyethyl starch, digoxin, and hyperbaric oxygen therapy (HBOT), but good human trials are needed [10,13,14,15,16,17].

HBOT has become the standard treatment for severe carbon monoxide poisoning, decompression illness (DCI), cerebral artery gas embolism, sudden sensorineural hearing loss (SSNHL), delayed radiation necrosis, infected wounds, especially for deep and chronic infections such as osteomyelitis, necrotizing soft tissue infections, and diabetic foot infections [18,19].

The main objective of this case report is to analyze the case of a patient with accidental ALP poisoning treated in the intensive care unit (ICU) of a local hospital in combination with hyperbaric oxygen therapy (HBOT). This case report is processed according to the CARE reporting guidelines [20].

## 2. Case Presentation

An almost-40-year-old man with a negative history of internal diseases was admitted to the ICU of a local hospital in the Central Moravian Region for suspected carbon monoxide poisoning. A gas boiler inside one of the buildings on the premises of the agricultural cooperative had been identified as a source of carbon monoxide. Elevated carbon monoxide levels of 50 ppm were detected on an autonomous detection device at the scene by members of the fire brigade. Two male subjects were found unconscious in one of the rooms. Both persons were from Eastern Europe and without valid work permits or valid health insurance. One of the patients experienced a cardiac arrest, was resuscitated, and later pronounced dead. The next person was the patient whose case we describe in this paper. The emergency medical crew took the patient to the nearest hospital, administering oxygen at a dose of 15 L/min. The patient’s state of consciousness improved during transport to the hospital. During admission to the hospital, the patient was somnolent, bradypsychic, and scored on the Glasgow Coma Scale (GCS) at 10–12. Spontaneous ventilation was adequate and no intubation or artificial or supportive pulmonary ventilation was required. Laboratory values at admission were as follows: lactate level 9.34 mmol/L, arterial blood gas analysis (ABG)-ph 7.27, pC02 3.2 kPa, p02 16.7 kPa, base deficit 13.8 mmol/L, serum osmolality 320 mmol/L, glycaemia 8.7 mmol/L, troponin I within normal range, and myoglobin level 213 ug/L. Carbonylhaemoglobin (COHb) level 0.5 (which was erroneously interpreted as 50%—see discussion) was reported by a laboratory technician on a call from the laboratory department.

Electrocardiography revealed an upward elevation of the ST segment in V2–V4. The medications used were as follows: normobaric oxygen therapy with a flow rate of 15 L/min, hydrocortisone 100 mg, and 1500 mL of balanced crystalloids (Plasmalyte, Baxter Czech Ltd., Prague, Czech Republic) were administered intravenously. After the primary treatment, taking into account the anamnestic data, clinical conditions, and available laboratory tests, this patient was referred to a telephone consultation with a physician on duty at the Centre of Hyperbaric Medicine, Ostrava. HBOT was immediately indicated and the patient was transferred to a multicenter hyperbaric facility.

HBOT therapy 2.5 ATA (250 kPa) was performed, 90 min of isopression, compression, and decompression at a rate of 6–8 kPa/min was administered. The patient was accompanied by medical personnel inside the hyperbaric unit throughout the procedure. HBOT was performed without complications. The patient became nauseous and was injected intravenously with 8 mg of ondansetron. Balanced crystalloid infusion (Plasmalyte, Baxter Czech Ltd., Prague, Czech Republic) was administered intravenously at a rate of 100 mL per hour. Upon the completion of HBOT treatment, the patient was immediately transported back to the local hospital in a stable condition. The patient was unable to be admitted for hospitalization because it was a weekend day, but mainly because it was during one of the highest levels of the COVID-19 pandemic, when there was a huge capacity problem to secure an intensive care bed at the Ostrava City Hospital. This was also the reason that only a single HBOT exposure was applied.

After being transferred back to the local hospital, a neurological examination, chest radiograph, and native brain CT were negative, and arterial blood gas analyses were normal. An increase in troponin was found on the second day, but without a clinical correlate, followed by a decrease in troponin. Echocardiography at the bedside was performed with the following result: left ventricle without kinetic disorder, without hypertrophy, and estimated left ventricular ejection fraction (EF) at 50%. Electrocardiography shows an upward increase in the ST segment in V2 of up to 2 mm. Furthermore, there was a laboratory insignificant increase in CRP with a gradual decrease, and an increase in liver transaminases (aspartate transaminase, AST 1.54 µcat/L). The patient was discharged from the hospital in a stable condition 4 days after admission.

In total, 48 h after the patient was treated with hyperbaric oxygen, the attending physician reported that it was probably not carbon monoxide intoxication, but phosphine intoxication. According to subsequent information from the representative of the Police of the Czech Republic, Gastoxin tablets containing ALP, releasing the toxic gaseous phosphine, which to some extent mimics the symptoms of carbon monoxide poisoning (with a considerable ability to bind to hemoglobin), were found in an adjacent building.

The exact cause of the intoxication was further investigated. Blood was drawn immediately after the man was admitted to the hospital, i.e., before the treatment began. For forensic purposes, the blood and urine of the survivor were re-analyzed and phosphine was found in the blood.

## 3. Discussion

The whole case was taken over by the Czech Republic Police on suspicion of committing a crime resulting in death, in cooperation with the Department of Forensic Medicine and Medical Law, Faculty of Medicine and Dentistry, Palacký University Olomouc, Czech Republic. For these reasons, the police have imposed a strict information embargo on all details of the case. This was released after the conclusion of the judicial process in 2022.

The cause of the phosphine poisoning was the fumigation of stored grain in an agricultural building adjacent to the building that served as a hostel. It was found that both buildings were connected by an underground corridor or a pipe through which the released poison gas penetrated.

It was also found that the detection device used by the firefighters evaluated phosphine as carbon monoxide; this was most likely caused by interference on the device’s detector (values around 50 ppm were mentioned). We found out by requesting information from fire-brigade rescuers that the multidetector GasAlert MicroClip XT (BW Technologies, Honeywell, Calgary, AB, Canada) was used during their rescue intervention. The question is why the acoustic alarm was activated when carbon monoxide was not present at the accident site. Since the physicochemical properties of phosphine are used in the semiconductor industry in the production of gallium phosphide, one of the most widely used semiconductors, and phosphine is used as an n-type dopant for doping polycrystalline silicon or certain capacitors [21], the theory is about whether this could have led to the alarm activation of the autonomous detector. This was the first event to have led to the misleading diagnosis of carbon monoxide intoxication.

The second misleading event was a report from the local hospital’s biochemistry laboratory on the level of carbonylhemoglobin, when a value of 0.5 was reported (percents were not mentioned). An inexperienced laboratory technician was on duty in the biochemical laboratory, and an inexperienced physician, a non-native with a certain language barrier, was on duty in the emergency department of the hospital. In our hospital, both arbitrary units and percents are commonly used for COHb values. Therefore, these values (arbitrary units and percents) were confused when the COHb value of 0.5 (arbitrary unit) was considered and interpreted as a value of 50%. Furthermore, this value theoretically corresponded to the severity described of the patient’s condition, as well as other critical laboratory values such as high values of arterial lactate or a base deficit in ABG.

Late information about the actual source of poisoning, the amount of confusing information, the severe condition of the patient, and last but not least the similar symptomatology of both diseases led us to the initiation of HBOT treatment in this patient. Note: As already mentioned, there was also a second person at the scene of the accident besides the patient whose case we describe, who unfortunately died from the accident. The body of the second man, who succumbed to phosphine intoxication, was sent for a forensic autopsy to the Institute of Forensic Medicine, where a comprehensive toxicological examination was performed as a part of the autopsy. The toxicology was focused on the proof of ethanol, addictive substances, drugs, and carbonylhemoglobin. Carbonylhemoglobin was investigated as a marker of carbon monoxide poisoning, as the police received information from the fire department that the presence of carbon monoxide was detected in the building where the men were found. The presence of carbon monoxide was recorded by a special gas-detection device. However, the toxicological examination of collected biological material did not show the presence of carbonylhemoglobin. After a toxicologist inspected the site where the men were found, a warehouse with grain that had been treated with phosphine was discovered. A subsequent and specifically focused toxicological analysis showed the presence of phosphine in the blood and lung tissue of the deceased man.

In total, 13 peer-reviewed articles were found and 63 articles were found in the gray literature. These covered 56 incidents from 1963 to 2019 for a total of 254 victims and 22 fatalities. There has been an increase in the number of reported cases in the last 20 years. Neurological and gastrointestinal symptoms are predominant and hospitalization is necessary in 80% of cases [5].

ALP is known as a suicide poison that can easily be bought. Its toxicity results from the release of phosphine gas as the tablet comes into contact with moisture. Phosphine gas mainly affects the heart, lungs, gastrointestinal tract, and kidneys. The symptoms and signs of poisoning include nausea, vomiting, restlessness, abdominal pain, palpitation, refractory shock, cardiac arrhythmias, pulmonary oedema, dyspnea, cyanosis, and sensory alterations. The diagnosis is based on clinical suspicion, a positive silver nitrate paper test for phosphine, and gastric aspirate and viscera biochemistry [1].

The severe toxicity of ALP particularly affects cardiac and vascular tissues, manifesting itself as profound and refractory hypotension, congestive heart failure, abnormal ECGs, myocarditis, pericarditis, and subendocardial infarction. Metabolic acidosis is again common, probably due to lactic acid caused by the blockage of oxidative phosphorylation and poor tissue perfusion. The severity of metabolic acidosis is also a prognostic indicator of ALP toxicity [10,22].

Strict precautions are required when using phosphine for the fumigation of cargoes and containers. Since symptoms are often vague, first responders should pay attention to the possible occurrence of acute phosphine intoxication, as it may be life-threatening. It is essential to implement in a strict way the existing legislation on an in-transit fumigation with phosphine. The training of the crew and good communication between the different actors during an in-transit fumigation (ship-owner, captain, fumigator, crew, and longshoremen) are the keys to a good prevention of accidents [5].

Container handlers can have an increased risk of neuropsychological symptoms, especially in the memory/concentration domain. Retail workers may also be at risk, but this requires confirmation in a larger study [6].

For treatment, experimental and clinical studies suggest the use of magnesium sulphate, melatonin, N-acetylcysteine, glutathione, sodium selenite, vitamin C and E, triiodothyronine, liothyronine, vasopressin, milrinone, Laurus nobilis, L-6-aminonicotinamide, boric acid, acetyl-L-carnitine, and coconut oil [23].

Meanwhile, some new antioxidants have been discovered and are expected to be used in the treatment of ALP poisoning. Furthermore, progress in intensive care has promoted technologies such as CRRT, IABP, and ECMO for the treatment of ALP poisoning, with reported success in alleviating severe toxicity [8].

The HBOT method in ALP intoxication was used in an experimental study in rats, where the authors studied the effects on survival time. All the animals exposed to ALP died within 5 days. The mean survival times of rats exposed to ALP without any intervention, treated with hyperbaric conditions via compressed air, and treated with hyperbaric conditions via pure O_2_, were 91 ± 1, 262 ± 8 (*p* < 0.001), and 276 ± 6 min (*p* < 0.001), respectively. HBOT may probably improve the survival time of the intoxicated rats with aluminum phosphide [17]. The prolongation of the survival of the animals poisoned with phosphoorganic compounds was also reported using HBOT at 3 ATA for 2–4 h [24].

## 4. Conclusions

The case was presented by the rescuers as well as healthcare professionals of the local hospital as carbon monoxide intoxication, which has a similar symptomatology to ALP intoxication. This case report describes the use of the HBOT for ALP intoxication. This was the first described case of using the HBOT method for ALP intoxication in the clinical medicine literature, although the HBOT indication itself in this case happened by coincidence. HBOT does not constitute standardized care in the management of ALP poisoning. Further studies must be undertaken to demonstrate the effectiveness of HBOT in treating patients with ALP poisoning.

## Data Availability

The data that support the findings of this study are available from the first author (M.H.) upon reasonable request.
